# Relative golf performance between men and women: A comparison of absolute differences in performance between players on the PGA and LPGA tours considering physiological and anthropometric sex-based differences

**DOI:** 10.1371/journal.pone.0323074

**Published:** 2026-05-11

**Authors:** Jemina Villanen, Arve Vorland Pedersen

**Affiliations:** Department of Neuromedicine and Movement Science, Faculty of Medicine and Health Sciences, Norwegian University of Science and Technology, Trondheim, Norway; West Virginia University, UNITED STATES OF AMERICA

## Abstract

Golf is an increasingly popular sport and presents fierce competition at the professional level. The Professional Golfers’ Association (PGA) Tour is the most prestigious professional men’s tour in golf, whereas the Ladies Professional Golf Association (LPGA) Tour is the equivalent for women. Among professionals, although women golfers play shorter courses than men, the rules and regulations for men and women are similar. In turn, differing requirements due to anthropometric and physiological sex-based differences may affect variables such as choice of club and playing strategy. Therefore, in our study, we aimed to identify differences in golf performance between men and women and to discuss whether the differences correspond with known physiological and anthropometric sex-based differences. We conducted a natural experiment, using publicly available data, comparing world class men and women golfers’ performances on their respective tours. Performance and course data were collected on all 50 PGA and 32 LPGA Tournaments in the 2021–2022 season and differences between the tours were identified. The lengths of the courses and holes on the LPGA Tour were approximately 90% of those on the PGA Tour. Overall, women had better driving accuracy than men but scored fewer eagles and rounds in the 60s. Anthropometric and physiological differences likely contribute to women’s adapted movement strategies during the swing and cause them to choose playing strategies that men do not, primarily in driving and approach shots. Altogether, our results suggest that women’s golf courses are likely not sufficiently scaled according to anthropometry or physiology. Thus, golf remains more demanding for women, which may, at least partly, explain their differences in performance compared with men.

## Introduction

Many sports have been scaled to better accommodate female biology—for example, by adjusting the rules and regulations—because it is well known that men are generally larger, faster, and stronger than women [1]. Among females, the average body size is approximately 90% of the size of an average male; however, the weight difference is greater, with women weighing approximately 79% what men weigh [2], possibly owing to different body composition. Women’s skeletal muscle mass is 61%–66% and their total fat-free mass is 68%–75% of men’s total skeletal muscle mass and fat-free mass, respectively [1]. Sex-based differences in muscle strength are also significant, more so in the upper than lower body. Women’s lower-body strength is roughly 60% of men’s, while in the upper body and arms, women’s strength is roughly 40–60% of men’s, depending on the body part measured [1]. For a detailed review see Nuzzo [1].

Scaling can involve factors such as the mass and/or size of the ball in team sports [3], different rules for body checking as in ice hockey [4], and/or the weight of thrown implements in throwing sports such as javelin [5]. However, many sports, including soccer, have the same rules and regulations for both sexes, and it has been argued that performance in such sports cannot be fairly compared between sexes without accounting for sex-based differences, if not scaling the rules and regulations [6].

In professional golf, though the rules and regulations are the same regardless of sex, the total length of golf courses in women’s professional tournaments is shorter than in men’s, likely to accommodate women’s shorter driving length [7]. The average yardage of the Ladies Professional Golf Association (LPGA) Tour courses is between 6,200 and 6,600 yards [8] but 7,200 yards for the men’s equivalent, The Professional Golfers’ Association (PGA) Tour courses [9]. Especially in recreational golf, the lengths of courses are further adjusted by the placement of tee boxes—that is, designated areas where the first shot for each hole is driven from. Courses usually offer several different tee options, thereby making the course’s length shorter or longer for players to accommodate their skill level. The LPGA Tour sets its own tees for each tournament, and the LPGA observes no guidelines or limitations for the lengths of women’s courses—for example, in relation to the length of men’s courses [10]. Even so, the LPGA often provides the longest tees on shorter courses, instead of making courses shorter by relocating tee box placements. According to Arthur et al. [10], the length of the longest tees on courses is determined by course architects and the course design, whereas shorter tees are more arbitrarily placed. Because women’s driving distance is shorter than men’s, playing shorter courses than men do arguably enables women to use the longer tees of courses and it is often assumed that, because of this, women can play the courses as they are designed to be played—for instance, in relation to negotiating different obstacles. After all, if hitting distances do not match a course’s design, then the nature of the challenges on the course change [7], along with the risk–reward element of challenging strokes [11].

However, as reported in Nichols’s [11] article in *Golfweek*, some professional women players argue that women’s courses remain, relative to their muscle strength, longer than men’s, which especially affects the choice of golf club used on approach shots on par 5 and par 4 holes, with par indicating the number of strokes that a player is expected to use to complete the hole. For additional evidence that women’s courses are, relatively, longer, regardless of being shorter for women, the mentioned players highlight that professional women players consistently have higher overall scores than men during competition rounds. Although driving distances on the PGA and LPGA Tours have steadily lengthened over the years, and the playing lengths of the courses therefore seem to increase [7], the distances have seemingly not been matched to meet women’s relative performance accordingly.

Within the extensive literature on golf performance, most studies have sought to identify physical factors contributing to golf performance, mainly focusing on the swing. Such studies have shown that golf club heads need high speeds to generate carry distance, which requires high levels of strength and muscle activation [12]. For instance, leg strength is correlated with golf performance [13] and, together with hip strength, helps to generate high levels of force toward the ground during the swing [14]. Low-handicap golfers are stronger, have more flexible hips and torsos, and tend to have more bench press strength [15]. In golf, a strong core is required to maintain a stable base for the lower extremities while rotating the torso and upper extremities [16], and rotary power in the torso helps to generate power during the swing [14]. Meanwhile, shoulder strength provides stability to the swing [14], and low-handicap golfers have more shoulder strength and shoulder flexibility than high-handicap ones [16]. On top of that, grip strength has been found to correlate with golf performance [13] and seems to be important in controlling the club when the club head is moving at high speeds [14].

Although most studies on golf performance have focused on strength, some have examined its other factors. Low-handicap golfers have demonstrated better single-leg balance than high-handicap ones, which may be associated with the shifting of weight during the swing and coping with varying lies of the ball on the course [16]. Low-handicap golfers have also been shown to have greater fat-free mass than high-handicap ones [15]. However, perhaps because golf is a low-intensity activity, there is no direct evidence of any relationship between cardiovascular condition and performance [12].

Upper arm and total arm lengths have been found to be longer among low-handicap golfers, who also had broader chests and arm girths [15]. In Wells et al.’s [13] study, arm length also correlated with golf performance, and the authors suggested that longer levers help to generate more force upon impact. In that study, anthropometric factors seemed to correlate more with players’ driver- and 5-iron performance as well as overall score but less with one’s short game (i.e., shorter strokes around the green). In general, there is limited evidence on the anthropometric and physiological factors related to one’s short game and putting, though height and limb lengths appear to correlate with putting, while core stability and strength might be important for the short game [13,17].

Most of the studies on golf performance, including the above-mentioned, have been conducted on men, and it therefore remains unclear how different the results would be for women. Nevertheless, it can be assumed that the physical requirements of golf are primarily the same regardless of sex. As a result, though the absolute requirements are similar they are, relatively, different between men and women. Along with the limited number of studies on women’s golf performance, studies on sex-based differences are lacking as well [18]. The long history of golf as a men’s sport, along with the rather slow narrowing of the sex gap [19], likely contributes to such androcentrism in research. However, some authors have nonetheless found differences in physiological demands and kinematics between men and women golfers [20–23].

Meanwhile, findings on sex-based differences in energy expenditure among golfers have been somewhat contradictory. Ilhan Odabas and Gercek [24] observed higher physiological and training loads and intensities among women, regardless of their shorter courses, whereas men seem to walk longer distances and faster [25]. Added to their greater body mass and height, men have greater arm spans [21,26], club head speeds [21,22,26], carry distances [22], and ball speeds [26].

At the same time, downswing duration is shorter among men than women [22,26], and women tend to have more shoulder and hip rotation at the top of their backswings [20,23], whereas men tend to flex their left knees more [20] and tilt their trunks forward more [23]. Regardless of such greater rotation, women have less uncoiling effect in their swings, with *uncoiling* being the effect produced when the torso acts as a spring between the hips and shoulders during the backswing, which endows considerable power to the swing [23]. Women have demonstrated significantly lower angular velocity and less elbow extension than men [23]. Men and women have also shown differences in kinematic variability and different movement strategies for the upper body during downswings [21]. Women, meanwhile, have a narrower stance width on average, even when adjusting for their lower stature [20]. According to club-related statistics representing PGA and LPGA players [27], women also have lower club speeds and ball speeds, lower maximal apex heights of shots, and shorter carry distances than men. However, because all the studies mentioned have focused on the kinematics of the swing, research on sex-based differences in other variables of performance has been limited.

Both the PGA and LPGA Tours offer a wealth of data on players’ performance, and a few studies have compared golfers’ performance on the PGA and LPGA Tours, albeit mostly in relation to scoring, success, and money wins. Moy and Liaw [28], for instance, compared performances on the PGA Tour, the LPGA Tour, and the Senior PGA Tour and concluded that LPGA players were far behind PGA players in sand saves (i.e., ability to recover a ball from a sand bunker), iron game (i.e., strokes with iron clubs), and their short game. Shmanske [29], who examined the effect of different skills on the earnings of professional golfers, identified putting and driving distance to be the most important skills for men, whereas putting and greens in regulation (i.e., par minus two strokes) were the most important for women. Shmanske directly compared men’s and women’s performance and concluded that no gender bias existed in the money wins: that the rewards were fair and based on absolute skill level. The prize money on the PGA Tour is approximately five times the prize money on the LPGA Tour [30,31], possibly due to such direct comparisons that ignore biological factors instead of making relative comparisons between sexes that would be fairer.

In a comparison of collegiate golfers, Callan and Thomas [32] discovered that men and women have different shot-making skills. In their study, men were better in every performance-related variable except fairways hit, thereby indicating that women’s drives landed more often on the fairway than men’s. Men improved more in performance from improving their sand saves, whereas women did so mostly by reaching greens in regulation and reducing the number of putts per round. More recently, Pfitzner et al. [31] found that the competition is stronger on the PGA Tour. LPGA Tour winners are more often top-ranked players, whereas PGA Tournaments sometimes have a winner who is not ranked among the top 200 in the world.

Despite golf’s growing popularity among women and the fact that women play it professionally across the world, even at the Olympic Games, the relatively limited amount of research on women’s golf and sex-based differences in the sport is somewhat surprising. The mentioned studies show, however, that differences in golf performance exist between men and women, though the reasons behind the differences have not been established. Women’s golf is frequently denigrated and portrayed as inferior to men’s [33], probably because people tend to compare what they see (i.e., absolute performance), not what they see while also considering who’s performing it and thus, they make absolute comparisons instead of relative ones [6]. Such comparisons are also reflected in the prize money, which may put women’s golf in a negative light as a less exciting sport. Given the physiological and anthropometric differences between men and women, however, there is reason to believe that much of the difference can be explained, which would suggest that sex-based differences in golf should be measured as relative performance. Therefore, in our study, we compared men’s and women’s golf performance by comparing available performance-related data and discussing that in relation to known average physiological and anthropometric differences between men and women, reported in previous studies, as well as some previously reported differences in men’s and women’s golf performance, in order to unearth relative differences in performance between men and women golfers.

## Methods

Data on golfers’ performance, together with the lengths of courses and their individual holes, were collected from tournaments played on the PGA and LPGA Tours in the 2021–2022 season, including data from all 50 PGA events and all 32 LPGA events. All collected data were publicly available and did not contain any sensitive information. No information about individual golfers is included in our results and they are presented anonymously as averages. Ethics approval was not applicable for this study, as the data were extracted from publicly available websites.

The data were organized in Microsoft Excel (version 2302), while IBM SPSS Statistics (version 28.0) was used for statistical analysis. Descriptive statistics were generated for all performance-related variables, as well as for course and hole lengths on both tours, and independent two-sample *t* tests were used to statistically examine differences between mean values on those variables on the PGA and LPGA Tours while applying a significance level of α < .05. Results are presented as means with standard deviations and ranges. Women’s performance as a percentage of men’s performance was also calculated for relevant variables in order to examine relative differences.

These results were discussed against generally well-known physiological and anthropometric differences between men and women, that have been reported by previous studies, and how those sex-based differences, in large part, could explain the differences in performance. Therefore, any physiological or anthropometric data on individual golf players on the tours were not collected, because it is the average differences between men and women in the general population that represent the sex-based differences on said variables; those which women golf players need to overcome in order to perform similar to men players.

### Performance

On their websites, both the PGA Tour [34] and LPGA Tour [35] provide performance-related data on different variables. For the PGA Tour, however, there are more data and different performance-related variables available than for the LPGA Tour. The PGA Tour has nine performance-related categories, each with 6–133 subcategories, while the LPGA Tour has eight such categories, each with 2–14 subcategories. Furthermore, for the PGA Tour, the data can be filtered by season or tournament, whereas data for the LPGA Tour are available only by season. Data for the 2021–2022 season were collected on all performance-related variables available for both the PGA and LPGA Tours and can thus be compared between both tours. For the variable of number of rounds under par, the LPGA Tour presents that number, as well as the same number as a percentage of all rounds; the PGA Tour, however, presents only the number of rounds under par. Because the actual number of rounds under par cannot be compared due to different numbers of total rounds played, we derived the number of total rounds for each PGA player from other statistics and calculated the percentage of rounds under par in order to create comparable units. Similar calculations were made for the variable of rounds in the 60s (i.e., rounds in the 60–69-stroke range). Furthermore, the LPGA Tour lists the number of birdies, eagles, and sub-par holes as an average per round, whereas the PGA Tour provides only the total number for those variables. Therefore, the average per round was calculated for the PGA Tour by dividing the total number of birdies, eagles, and sub-par holes by the number of total rounds for each player. All variables, along with their definitions, are listed in Table 1.

**Table 1:** Variables examined and their definitions according to the Professional Golfers’ Association (PGA).

Variable	Definition
**Driving distance**	Average driving distance in yards
**Driving accuracy**	Percentage of times that the drive ends up in the fairway
**Greens in regulation**	Percentage of times that the player reaches the green in par minus two strokes
**Putts per greens in regulation**	Average number of putts per greens that are hit in regulation
**Putting average**	Average number of putts per hole
**Sand saves**	Percentage of times that the player was able to get the ball in the hole with two or fewer shots from a greenside bunker
**Scoring average**	Average number of strokes per completed round
**Rounds under par**	Percentage of the number of rounds that the player was able to obtain an under-par result
**Birdies per round**	Number of birdies (i.e., one under par) per round
**Eagles per round**	Number of eagles (i.e., two under par) per round
**Rounds in the 60s**	Percentage of the number of rounds with a score in the 60–69-stroke range
**Sub-par holes per round**	Number of holes per round with a score less than par
**Par 3 scoring average**	Average number of strokes on par 3 holes
**Par 4 scoring average**	Average number of strokes on par 4 holes
**Par 5 scoring average**	Average number of strokes on par 5 holes
**Strokes gained total**	Average number of strokes per round that the player was better or worse than the rest of the field (i.e., all players playing the same course in the same event on the same day)
**Strokes gained tee to green^1^**	Average number of strokes that the player was better or worse by compared with the rest of the field in parts of play other than putting
**Strokes gained off the tee^2^**	Average number of strokes that the player was better or worse by compared with the rest of the field off the tee (i.e., driving) on par 4 and par 5 holes
**Strokes gained approaching the green^3^**	Average number of strokes that the player was better or worse by compared with the rest of the field in approach shots
**Strokes gained around the green^4^**	Average number of strokes that the player was better or worse by compared with the rest of the field in ‘around the green’ play
**Strokes gained putting^5^**	Average number of strokes that the player was better or worse by compared with the rest of the field in putting

^1^Calculated, by the PGA, by subtracting the player’s strokes gained putting from the player’s strokes gained total.

^2^Calculated, by the PGA, by measuring the number of the players’ off-the-tee strokes from specific distances or locations against a statistical baseline. The strokes gained or lost off the tee for that round is the sum of those values for all holes, minus the field’s average number of strokes gained or lost for the round. Strokes gained for each round are then added together and divided by total rounds played.

^3^Calculated, by the PGA, by measuring the number of strokes in the players’ approach to the green from specific distances or locations against a statistical baseline. The strokes gained or lost approaching the green for that round is the sum of those values for all holes, minus the field’s average strokes gained or lost for the round. Strokes gained for each round are then added together and divided by total rounds played.

^4^Calculated, by the PGA, by measuring the number of strokes around the green from specific distances or locations against a statistical baseline. The strokes gained or lost around the green for that round is the sum of those values for all holes, minus the field’s average strokes gained or lost for the round. Strokes gained for each round are then added together and divided by total rounds played.

^5^Calculated, by the PGA, by measuring the number of putts from specific distances or locations against a statistical baseline. The strokes gained or lost putting for that round is the sum of those values for all holes, minus the field’s average strokes gained or lost for the round. Strokes gained for each round are then added together and divided by total rounds played.

Data for the study were available for 193 players on the PGA Tour and 163 players on the LPGA Tour, except for a few variables on both tours in which more players were listed. The data were organized such that only the 163 and 193 players listed for all relevant variables were included in analysis; thus, the data used in our analysis contained 163 women and 193 men. The sole exception was the variable of eagles on the LPGA Tour, for which only 149 players were listed. The LPGA Tour presumably listed only those players who scored at least one eagle during the season, because the list contained no players with zero eagles, whereas the PGA Tour also listed those players who had scored zero eagles. Thus, for analysis, we calculated an additional average for women by adding the missing 14 players without any eagles.

Added to the analysis including all players, similar statistics were calculated when including only the 20 best-ranked players on each performance-related variable on each tour. The statistics for all variables involving strokes gained were calculated only for the top players, because values for strokes gained present performance in relation to other players, such that the mean values of all players are not informative.

### Courses

The lengths of each course and hole were collected for all 50 tournament events on the PGA Tour and all 32 tournament events on the LPGA Tour for the 2021–2022 season. For the PGA Tour, those data are available on the PGA’s website [36] for all courses and all their holes, except for three courses, for which the information was collected from the respective events’ official websites [37–39]. The LPGA Tour’s website offers very limited data on the courses aside from providing the total length of each course. More detailed course-related information, however, was available on the LPGA Tour Online Media Center [40].

Statistical analyses were performed for differences in course lengths using the total lengths of all courses played during the season. To analyze the par 3, par 4, and par 5 holes, we collected the distances of all holes on each course, listed them in three groups based on par, and calculated statistics for par 3, par 4, and par 5 holes for both tours separately.

## Results

### Performance

Table 2 presents data representing the LPGA players’ and PGA players’ performance, *p* values for differences between them, and differences presented as the mean LPGA players’ values as a percentage of the mean PGA players’ values. LPGA performance in relation to PGA performance was poorer for the rounds in the 60s, eagles per round, rounds under par, and driving distance. Women also had fewer sand saves, birdies per round, and sub-par holes per round. The percentage of rounds under 70 strokes for LPGA players was approximately 67% of the PGA players’ corresponding percentage, while the number of eagles was 70%. When adding the 14 LPGA players without any eagles (see Methods for an explanation why), the average number of eagles was 0.06, such that women’s performance was only 60% of the men’s. The percentage of rounds under par for women was approximately 88% of the men’s, while their driving distance was approximately 86% of men’s. Women’s performance in relation to men was approximately 90% for sand saves, 92% for sub-par holes per round, and 93% for birdies per round. The only variable for which women were noticeably better was driving accuracy, with their performance being approximately 22% better than men’s. The relative LPGA player greens in regulation percentage was slightly higher than PGA players’, although they had slightly more putts on the greens that were hit in regulation. Even so, those differences were slight. The putting average for women was 0.06 putts more than men’s and their scoring average 1.21 strokes more. Men and women had similar performance on par 3 holes, but the difference was greater on par 4 holes and greatest on par 5 holes. All performance differences between men and women golfers on the examined variables were statistically significant except for the par 3 scoring average.

**Table 2:** Performance of players in the Ladies Professional Golf Association (LPGA) Tour and Professional Golf Association (PGA) Tour on comparable variables.

Variable	LPGA	PGA	p (α = .05)	Relative performance
*Driving distance (yards)*	257.12 ± 9.27 (235.11–279.26)	300.49 ± 8.68 (277.40–321.40)	<.001	85.57%
*Driving accuracy (%)*	73.62 ± 5.91 (56.20–87.20)	60.12 ± 4.97 (48.18–73.95)	<.001	122.46%
*Greens in regulation (%)*	68.74 ± 4.02 (57.00–77.70)	66.30 ± 2.40 (57.82–72.29)	<.001	103.68%
*Putts per greens in regulation*	1.81 ± 0.04 (1.72–1.92)	1.76 ± 0.02 (1.68–1.82)	<.001	102.84%
*Putting average*	1.67 ± 0.03 (1.58–1.76)	1.61 ± 0.02 (1.54–1.68)	<.001	103.73%
*Sand saves (%)*	45.23 ± 7.73 (19.23–66.25)	50.09 ± 6.38 (30.26–67.09)	<.001	90.30%
*Scoring average*	71.60 ± 1.11 (68.99–74.46)	70.39 ± 0.61 (68.84–72.03)	<.001	101.72%
*Rounds under par (%)*	45.13 ± 14.66 (11.11–76.47)	51.16 ± 7.69 (25.00–69.66)	<.001	88.21%
*Birdies per round*	3.27 ± 0.45 (2.17–4.51)	3.53 ± 0.30 (2.83–4.53)	<.001	92.63%
*Eagles per round*	0.07 ± 0.04 (0.01–0.22)	0.10 ± 0.04 (0.00–0.24)	<.001	70.00%
*Rounds in the 60s (%)*	25.57 ± 11.34 (2.94–57.65)	38.31 ± 8.39 (16.07–60.61)	<.001	66.74%
*Sub-par holes per round*	3.33 ± 0.47 (2.22–4.56)	3.63 ± 0.32 (2.85–4.70)	<.001	91.74%
*Par 3 scoring average*	3.05 ± 0.07 (2.89–3.27)	3.05 ± 0.04 (2.96–3.18)	.398	100.00%
*Par 4 scoring average*	4.07 ± 0.07 (3.94–4.31)	4.03 ± 0.04 (3.94–4.12)	<.001	100.99%
*Par 5 scoring average*	4.75 ± 0.08 (4.56–4.97)	4.63 ± 0.07 (4.41–4.85)	<.001	102.59%

*Note.* Performance presented as M ± SD (min.–max.), with relative performance being performance of the LPGA as a percentage of the performance of the PGA.

The performance of the 20 best LPGA and PGA players on each performance-related variable is presented in Table 3. The differences in mean driving distance and mean driving accuracy were similar to the differences of means when comparing all players. The performance of LPGA players in relation to that of PGA players on greens in regulation, putts per greens in regulation, and sand saves were slightly better for the top players than for all players, whereas the difference in putting average was similar. The difference in the scoring average was smaller among the top players: only 0.5 strokes compared with 1.2 strokes when including all players. The top 20 LPGA players’ rounds under par percentage was approximately 9% higher than that of the top 20 PGA players. Meanwhile, sex-based differences in eagles per round and rounds in the 60s were smaller among the top players, with women’s performance at approximately 90% of men’s. In birdies per round and sub-par holes per round, the top 20 LPGA players reached approximately 99% of the PGA players’ performance, and those differences were not statistically significant. Par 3, par 4, and par 5 performance followed a similar pattern as when all players were analyzed; however, the top 20 LPGA players’ par 3 performance was slightly better than the top 20 PGA players’ performance, and par 4 performance did not significantly differ between men and women.

**Table 3:** Performance of the 20 best players in the Ladies Professional Golf Association (LPGA) Tour and 20 best players in the Professional Golf Association (PGA) Tour on comparable variables.

Variable	LPGA	PGA	p (α = .05)	Relative performance
*Driving distance (yards)*	272.44 ± 2.98 (268.05–279.26)	315.91 ± 3.36 (310.80–321.40)	<.001	86.24%
*Driving accuracy (%)*	83.47 ± 2.10 (80.40–87.20)	68.81 ± 1.93 (66.83–73.95)	<.001	121.31%
*Greens in regulation (%)*	75.00 ± 1.40 (73.30–77.70)	70.42 ± 0.99 (69.12–72.29)	<.001	106.50%
*Putts per greens in regulation*	1.75 ± 0.01 (1.72–1.77)	1.72 ± 0.01 (1.68–1.73)	<.001	101.74%
*Putting average*	1.62 ± 0.02 (1.58–1.63)	1.57 ± 0.01 (1.54–1.58)	<.001	103.18%
*Sand saves (%)*	57.90 ± 3.09 (54.29–66.25)	61.77 ± 2.66 (58.23–67.09)	<.001	93.73%
*Scoring average*	69.80 ± 0.32 (68.99–70.20)	69.30 ± 0.28 (68.84–69.63)	<.001	100.72%
*Rounds under par (%)*	69.26 ± 2.72 (65.39–76.47)	63.70 ± 2.70 (60.00–69.66)	<.001	108.73%
*Birdies per round*	4.06 ± 0.16 (3.85–4.51)	4.11 ± 0.16 (3.95–4.53)	.313	98.78%
*Eagles per round*	0.15 ± 0.03 (0.12–0.22)	0.17 ± 0.02 (0.14–0.24)	.003	88.24%
*Rounds in the 60s (%)*	46.85 ± 4.34 (40.48–57.65)	52.59 ± 3.59 (48.61–60.61)	<.001	89.09%
*Sub-par holes per round*	4.16 ± 0.15 (3.90–4.56)	4.22 ± 0.18 (4.06–4.70)	.260	98.58%
*Par 3 scoring average*	2.94 ± 0.03 (2.89–2.97)	2.99 ± 0.01 (2.96–3.01)	<.001	98.33%
*Par 4 scoring average*	3.97 ± 0.02 (3.94–4.00)	3.96 ± 0.01 (3.94–3.98)	.077	100.25%
*Par 5 scoring average*	4.61 ± 0.03 (4.56–4.64)	4.50 ± 0.04 (4.41–4.54)	<.001	102.44%
*Strokes gained total*	1.71 ± 0.28 (1.31–2.44)	1.47 ± 0.28 (1.07–2.12)	.011	116.33%
*Strokes gained tee to green*	1.30 ± 0.45 (0.93–2.81)	1.30 ± 0.21 (1.03–1.77)	.976	100.00%
*Strokes gained off the tee*	0.68 ± 0.12 (0.49–0.89)	0.64 ± 0.15 (0.45–1.02)	.342	106.25%
*Strokes gained approach the green*	0.98 ± 0.37 (0.62–2.09)	0.70 ± 0.14 (0.49–1.06)	.003	140.00%
*Strokes gained around the green*	0.36 ± 0.19 (0.24–1.10)	0.41 ± 0.06 (0.33–0.52)	.293	87.80%
*Strokes gained putting*	0.83 ± 0.22 (0.62–1.36)	0.59 ± 0.13 (0.45–0.85)	<.001	140.68%

*Note.* Performance presented as M ± SD (min.–max.), with relative performance being performance of the LPGA as a percentage of the performance of the PGA.

On average, the top 20 LPGA players gained approximately 16% more total strokes than the top 20 PGA players. The top LPGA players also gained approximately 40% more strokes in approaching the green and in putting than the top PGA players. Strokes gained was poorer for the top LPGA players only around the green, where they averaged approximately 88% of the top 20 PGA players’ average; however, the difference was not significant. The strokes gained tee to green were similar between men and women as well.

### Courses

None of the LPGA Tournaments were played on the same course as the PGA Tournaments, and the LPGA Tournament courses were generally shorter than the PGA’s. The mean lengths and differences in the LPGA versus PGA courses and holes appear in Table 4. On average, the course length on the LPGA Tour was 737 yards shorter, thus approximately 90% of the average PGA course. The longest LPGA course was only 66 yards longer than the shortest PGA course, and there was an 871-yard difference between the longest courses of the respective tours. The par 3 holes were 26 yards shorter for women on average, whereas par 4 holes were 47 yards shorter and par 5 holes 54 yards shorter. However, the shortest par 3 hole was slightly shorter on the PGA Tour than the corresponding one on the LPGA Tour. Although the lengths of the LPGA holes were approximately 90% of the PGA holes, the difference decreased slightly as par increased. The difference was biggest on par 3 holes, where the LPGA Tour length was approximately 87% of the PGA Tour length. All differences in mean lengths were statistically significant.

**Table 4:** Lengths of courses and holes on the Ladies Professional Golf Association (LPGA) and Professional Golf Association (PGA) tours.

	LPGA	PGA	p (α = .05)	Relative length
*Course length (yards)*	6,566 ± 158 (6,190–6,894)	7,303 ± 205 (6,828–7,765)	<.001	89.91%
*Par 3 length (yards)*	170 ± 21 (110–234)	196 ± 27 (106–265)	<.001	86.73%
*Par 4 length (yards)*	388 ± 31 (290–442)	435 ± 46 (296–550)	<.001	89.20%
*Par 5 length (yards)*	519 ± 30 (450–585)	573 ± 33 (503–677)	<.001	90.58%

*Note.* Lengths of the courses and holes in the LPGA and the PGA events presented as M ± SD (min.–max.), with relative lengths being the lengths for the LPGA as a percentage of the lengths for the PGA.

## Discussion

Our study was the first to compare relative differences between men’s and women’s performance in professional golf. Our analysis included major performance-related variables and lengths of courses, which revealed some clear differences in performance, as well as in scaling needs, if women’s golf is expected to be similar to men’s. However, the purpose of our study is not to argue for scaling down women’s golf courses, but to point to the differences in anthropometry and physiology, that women golfers need to adapt to.

The most striking sex-based differences in golf performance were in rounds in the 60s and eagles, followed by driving distance and driving accuracy (Table 2), which could suggest that the courses are, relatively, longer for women and that women may have developed, along with different swing kinematics found in previous studies, playing strategies that differ from men’s strategies in order to score better on the course.

Driving distance is indeed one of the most discussed differences between men’s and women’s golf, if not one of the most examined topics in golf. According to our results, professional women players’ driving distance is approximately 86% of men’s (Table 2), though women’s strength is generally only 40%–60% of men’s [1]. If driving distance was determined by strength only, then women should not be able to achieve such long relative driving distances, which suggests that women have made other adaptations to their swings to compensate for the lack of strength. As previous research has shown, there are indeed sex-based differences in swing kinematics. Although women reach approximately 85% of men’s club head speed [27], they do so by using different movement strategies [21]. With only 60% the hip and trunk strength of men [1], women cannot generate as much rotary power [14] for the swing and likely have to compensate with greater rotation [20,23], which may consequently affect the uncoiling effect [23] and downswing duration [22,26]. Because leg strength seems to be important in generating force toward the ground [14] and because women have only 60% of that strength compared to men [1], they might be using their legs differently and therefore flex their knees less than men [20]. Other findings indicate the different use of legs in golf between men and women [13]. The narrower mean stance found among women [20] might affect balance, which has been suggested to be important during the swing [16]. Women’s shoulder and elbow strength, at approximately half of that of men [1], likely contributes to their lesser elbow extension [23] and might provide less shoulder stability, which seems to be important for performance [16]. Differences in the angular velocities of the wrist and elbow [23] are likely a consequence of the fact that all parts of women’s bodies generate less power than men’s, especially the upper body. Grip strength is also important in controlling the golf club [14], and women have approximately 60% of men’s grip strength [1]. However, with a lower club head speed, they might not need more of their maximal strength than men do theirs in controlling clubs.

As our results show, LPGA courses, as well as their par 3, 4, and 5 holes, are approximately 90% of the length of the PGA’s (Table 4), and the difference is close to the difference in driving distance. Although those differences may seem to suggest reasonable scaling, they do not consider the lengths of approach shots, which may vary considerably between men and women. The best way to investigate that possibility would have been to collect data on the approach shot distances and clubs used, but such data were unavailable. The matter can be roughly estimated, however, by investigating average hole and stroke lengths.

Among LPGA players, the average driving distance of 257 yards (Table 2), average par 4 length of 388 yards, and average par 5 length of 519 yards (Table 4) indicate an average approach shot distance of 131 yards on par 4 holes and 262 yards on par 5 holes. According to the average carry distances of LPGA players with different clubs [27], women would have to hit the approach shot with an 8-iron on par 4 holes and could not reach the green on the second shot on par 5 holes, even if they took another stroke with a driver. A similar calculation for the PGA Tour reveals that men would hit the approach shot on par 4 holes with a pitching wedge and on par 5 holes could reach the green with a 3-wood with an average carry distance of 243 yards when taking the roll into account. With similar calculations for the shortest par 5 holes (Table 4), women would need a 3-wood to reach the green on the second stroke, whereas men could use a 4-iron.

Likely for that reason, our analysis revealed two variables with far greater sex-based differences than driving distance: rounds in the 60s and eagles per round (Table 2). Those variables have not been included in past studies, perhaps because they are not viewed as variables that describe performance itself. In any case, the finding highlights problems with courses that are, relatively, longer for women that have been underscored by players [11]. If par 5 holes are, relatively, longer, then scoring an eagle would be more difficult for women, and consequently, the odds of scores under 70 strokes. Moreover, differences in hole length seem to decrease and differences in scoring average to increase, albeit only slightly, the higher the par score (Tables 2 and 4). On par 5 holes, players usually need two to three longer strokes to reach the green. Considering that the relative sex-based difference in stroke length for all clubs is rather similar to that of drivers [27], the relative difference in hole lengths should instead increase with as par score increases, especially if the aim was to enable women to play the holes in a way similar to men (e.g., in terms of club choices).

Our results, when compared with published ones, show that the driving distance has increased on both tours, although slightly more on the PGA Tour, which has caused that sex-based difference to be slightly greater than before. However, driving distances are constantly increasing on both tours, which has caused problems with course designs on men’s tours, for the courses may be too short to be played as designed [7]. Thus, instead of LPGA courses being too long, PGA courses might be too short. However, according to professional woman players, women’s golf is less interesting to watch—and to play—due to the lack of drivable par 4 holes and reachable par 5 holes and the risk–reward element that those kinds of holes provide [11]. Therefore, when hitting a drive, women may not be able to take the same risks as men—for instance, when considering the different obstacles. Instead, they might have to approach the green by first hitting the ball on the fairway, which could explain why women had approximately 20% better driving accuracy than men in our results (Table 2).

The difference in driving accuracy seems to have changed over the years. In earlier studies, driving accuracy was better among men [28,29]; however, in the 2013 season, women outperformed men by 15% [31]. When our findings are compared with published results, PGA players’ driving accuracy has also decreased over the years, whereas LPGA players’ has increased. Added to riskier shots, women overtaking men’s driving accuracy could be due to the rising importance of driving distance [7], which urges men to deploy more power to their drives at the expense of accuracy. According to McGinnis and Gentry [19], women seem to be more concerned with distance than men, but coaches tend to encourage women to worry more about accuracy than distance. If such were the general trend in women’s golf, then it could explain those differences. Beyond that, women may compensate for the limits of their physical capacity to drive the ball even farther by increasing their accuracy.

Differences in performance in the other variables were smaller but could suggest slightly different sex-based playing strategies. Our results reveal that the average women’s performance in putting average and putts per greens in regulation was slightly poorer than men’s, whereas the LPGA’s average performance in greens in regulation was slightly better than the PGA’s (Table 2), especially when comparing only the best players (Table 3). Greens in regulation was also one of the most important predictors of success for the LPGA Tour in previous studies [28,29,31,32].

Shmanske [29] has suggested that reaching the green in regulation could be more difficult for men than women because of the extra length of a PGA Tour course. However, based on stroke lengths, it is women who have, relatively, longer courses; thus, they would presumably have lower greens in regulation percentage than the men, which according to our results is not the case. At the same time, if a green is reached in regulation, then it simply means that the ball is on the green but can be farther away from the hole than a ball lying next to the green, depending on the pin placement. If women have to hit approach shots with longer clubs, then it provides less accuracy for where the ball comes to lie on the green; thus, the goal can be to simply hit the green. Men, by contrast, can target the pin more accurately with shorter clubs. That seeming advantage, however, can mean more risky shots, and the ball might end up outside the green but still rather close to the hole. When a player hits the ball to the green from close to the green, landing it closer to the hole is easier, which results in fewer putts. Pfitzner et al. [31] have also highlighted that higher greens in regulation percentage on the LPGA Tour and fewer putts on the PGA Tour are related, for PGA players probably have more one-putt greens due to greens missed in regulation. Our results that top women gain more strokes than men in approaching the green and in putting, whereas the top men gain more strokes around the green (Table 3), together with the fewer number of putts among men (Tables 2 and 3), further supports that view. Although the top players in terms of individual performance-related variables are not necessarily the top players overall, the described dynamic could mean that those are important skills considering success on the tour.

Although putts per green in regulation considers only putts after the green is hit in regulation, women still seem to slightly underperform men on that variable (Table 2). Shmanske [29], who used the variable to make predictions, concluded that the payoff for improving putting skills is higher for men, which may explain why men are better putters, because there are no apparent sex-based reasons for that. However, as mentioned, even if the green has been reached in regulation, men could have used clubs with better accuracy and thus ended up closer to the hole due to the shorter approach shot, which would make it easier to finish the hole with fewer putts.

While putting does not require as much strength as longer strokes, anthropometric and physiological factors can explain part of the differences in putting performance as well. It stands to reason that women use more of their maximal strength than men on shorter strokes or putts with the same distance, which might affect accuracy. Although the relationship between anthropometric and physiological factors and putting performance in previous studies is not clear, core strength and height, along with limb length, are suggested to be important [13,17], which could contribute to women’s slightly poorer performance. Wells et al. [13] found that forearm strength correlated with putting performance among women but not men, which suggests different sex-based muscle recruitment patterns. In turn, those patterns could mean that women have different kinematics not only in the full swing but in putting as well. The correlation between stature and putting could also relate to a taller person’s ability to see the putting line better.

Sand save percentage among women has increased since Moy and Liaw’s [28] study, which demonstrated that LPGA players lag far behind PGA players in performance. In our study, women attained approximately 90% of men’s sand save performance (Table 2). The difference might relate to poorer putting performance, for a sand save is recorded only if the player manages to putt the ball into the hole after the bunker shot. However, strokes from the bunker require more strength, thereby making the relative requirements for men and women different, which might affect the accuracy and, in turn, the length of the subsequent putt.

Although we found that women had shorter driving distances and score fewer eagles and rounds in the 60s, the differences in birdies, rounds under par, sub-par holes, and scoring average were not similarly large (Table 2). Those results again suggest that women have adapted their play to obtain those scores in ways possibly different from men. In view of previous studies, the difference in scoring averages between the PGA and LPGA Tours decreased from two strokes [28,29] to 1.5 strokes in 2013 [31] and to 1.2 strokes in our study (Table 2). Women’s performance seems to be improving in most variables, though the increase in driving distance and all other variables seems insufficient to increase the performance in eagles and under 70 scores and thus to equal men’s scoring average, which again suggests that courses are, relatively, longer for women.

Our findings support Pfitzner et al.’s [31] finding that the competition is tougher on the PGA Tour considering average performance. When analyzing differences between the top 20 PGA and LPGA players, the LPGA’s performance is much more similar, if not better, than the PGA’s (Table 3), which indicates that the best players on the LPGA Tour are more concentrated at the top than on the PGA Tour. The findings indicating that the top LPGA players also gain more strokes than the top PGA players (Table 3) and the larger standard deviations for all performance-related variables among women (Table 2) further support that trend.

According to Shmanske [29], differences other than course length might exist between the PGA and LPGA courses, including fairway width, green size, and bunker depth. Those other factors, though beyond the scope of our study, warrant further research. The purpose of the discussion above is to show that while the absolute golf performance between men and women players is different, the relative performance (i.e., the performance relative to the anthropometric and physiological capacity of the male vs. the female body) may not be that different. Closer investigation into different shot distances, clubs used, and trends in risk-taking would reveal more information on sex-based differences in playing strategies. In general, there is a lack of studies analyzing women’s performance in golf and, studies on physiological variables suggested to be important in golf that we also examined primarily represent men golfers. Moreover, different variables may be more important for women than for men, meaning that additional research on women golfers is required. Last, because the availability of data for women was poorer than for men, collecting and publishing data more extensively could encourage additional research on the topic.

## Conclusion

In professional golf, courses and holes seem to be, relatively, longer for women, which prevents women from being able to play the courses similarly to men. Given our results, and likely as a consequence of the relative differences in hole lengths, it seems that men and women employ slightly different playing strategies to achieve good scores on competition rounds. The scaling of women’s golf, according to anthropometry and physiology, does not seem to be sufficient, which makes golf more demanding for women than men, especially on longer holes.
